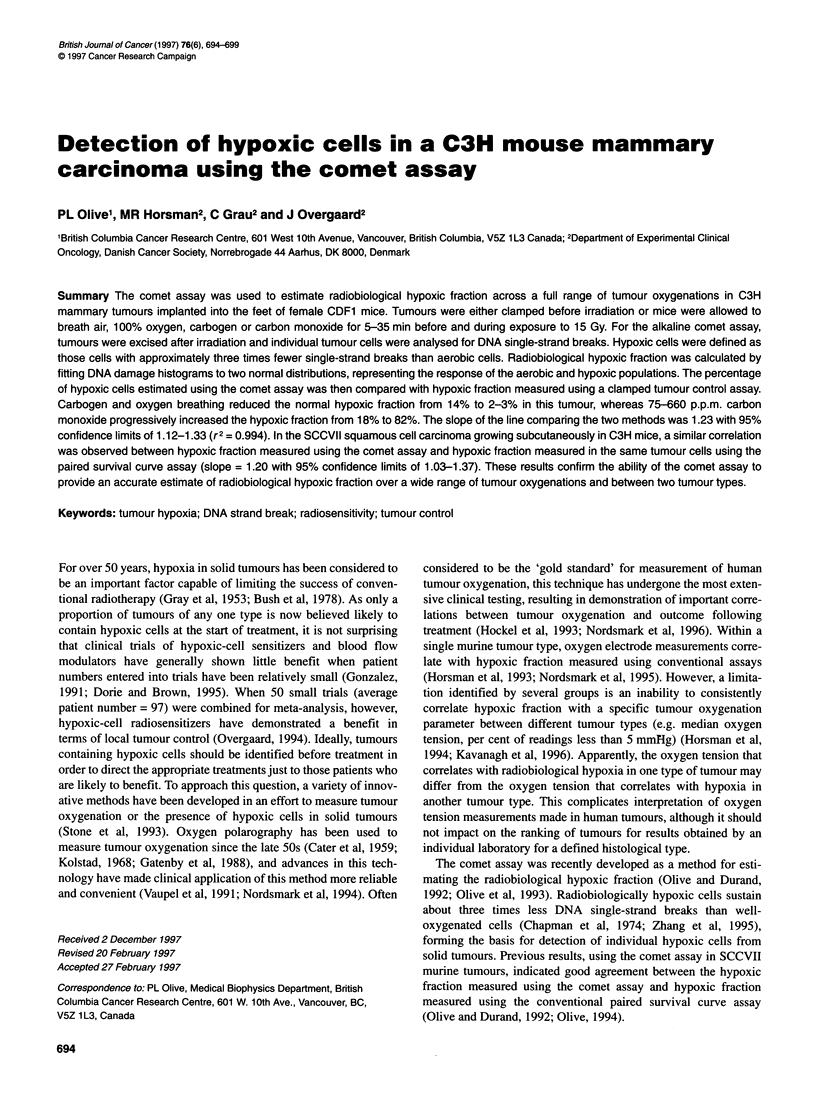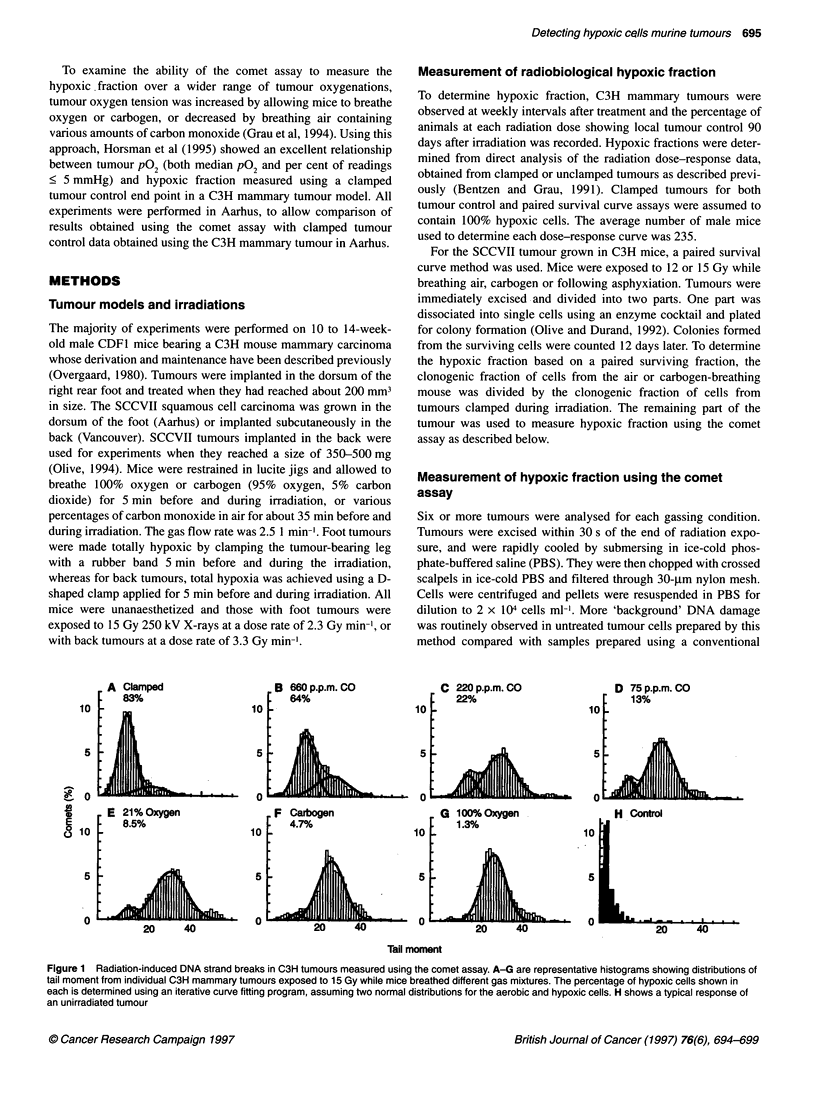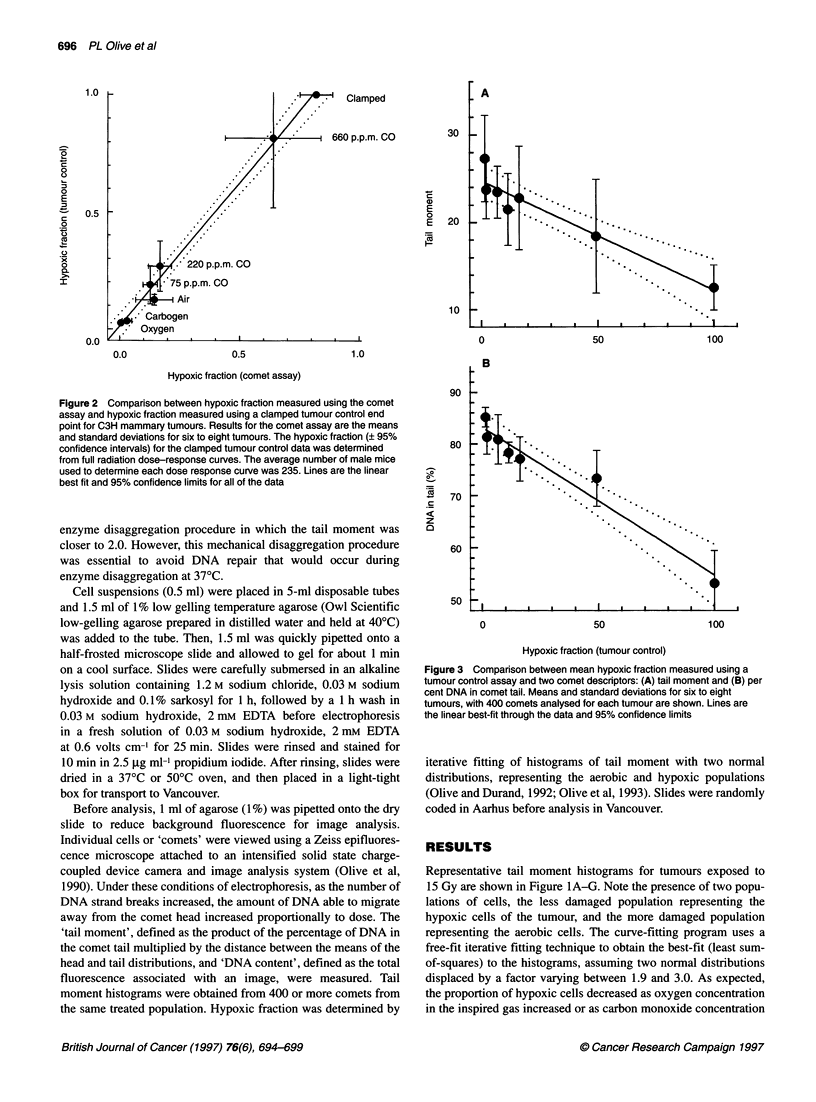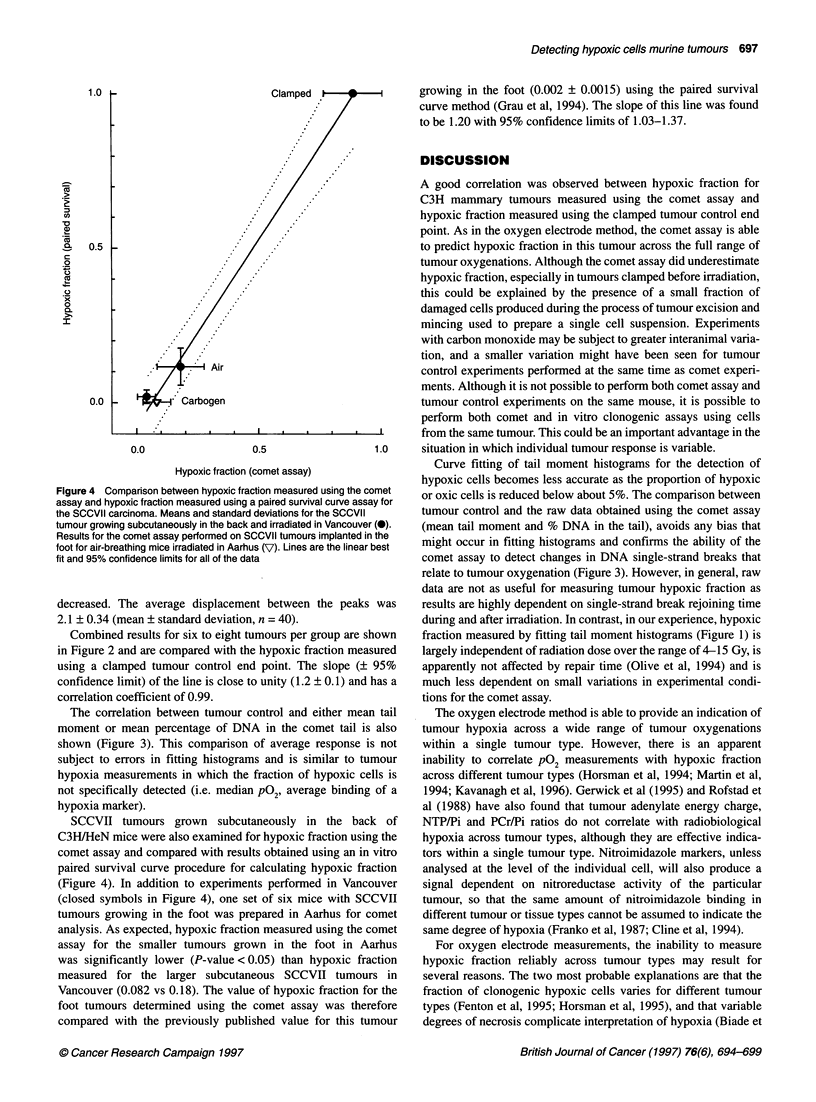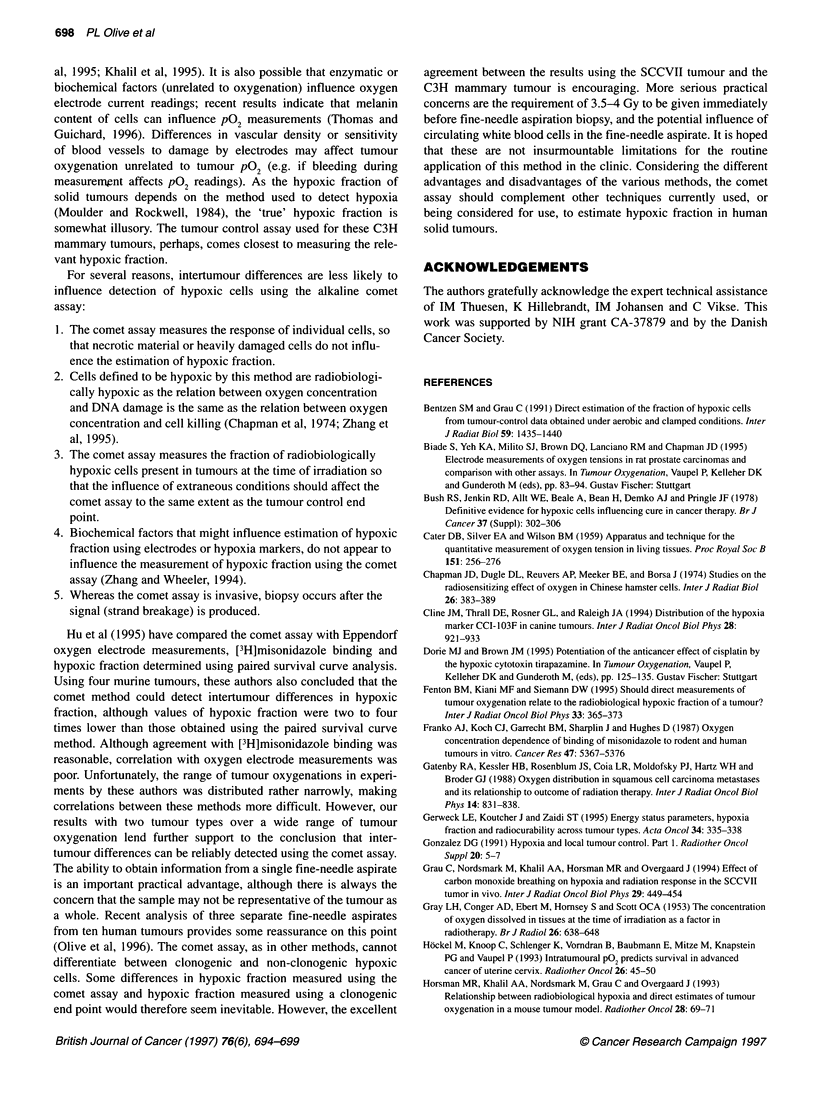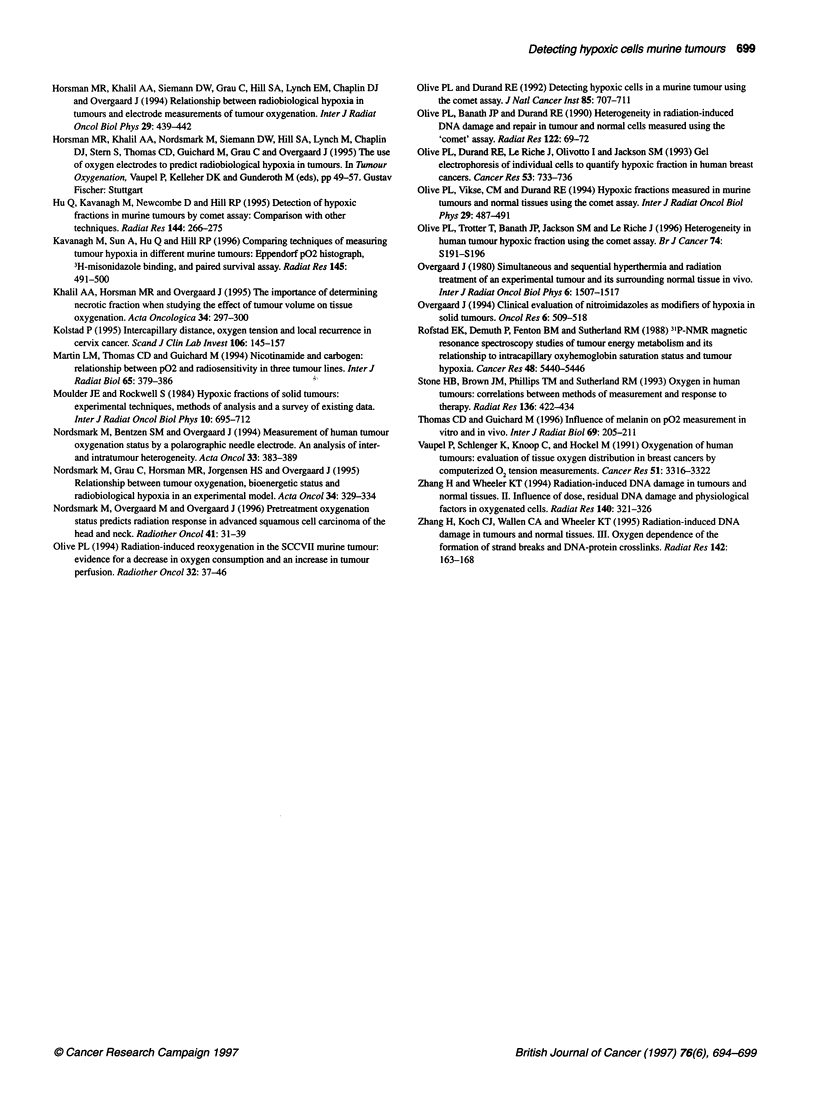# Detection of hypoxic cells in a C3H mouse mammary carcinoma using the comet assay.

**DOI:** 10.1038/bjc.1997.448

**Published:** 1997

**Authors:** P. L. Olive, M. R. Horsman, C. Grau, J. Overgaard

**Affiliations:** British Columbia Cancer Research Centre, Vancouver, Canada.

## Abstract

The comet assay was used to estimate radiobiological hypoxic fraction across a full range of tumour oxygenations in C3H mammary tumours implanted into the feet of female CDF1 mice. Tumours were either clamped before irradiation or mice were allowed to breath air, 100% oxygen, carbogen or carbon monoxide for 5-35 min before and during exposure to 15 Gy. For the alkaline comet assay, tumours were excised after irradiation and individual tumour cells were analysed for DNA single-strand breaks. Hypoxic cells were defined as those cells with approximately three times fewer single-strand breaks than aerobic cells. Radiobiological hypoxic fraction was calculated by fitting DNA damage histograms to two normal distributions, representing the response of the aerobic and hypoxic populations. The percentage of hypoxic cells estimated using the comet assay was then compared with hypoxic fraction measured using a clamped tumour control assay. Carbogen and oxygen breathing reduced the normal hypoxic fraction from 14% to 2-3% in this tumour, whereas 75-660 p.p.m. carbon monoxide progressively increased the hypoxic fraction from 18% to 82%. The slope of the line comparing the two methods was 1.23 with 95% confidence limits of 1.12-1.33 (r2 = 0.994). In the SCCVII squamous cell carcinoma growing subcutaneously in C3H mice, a similar correlation was observed between hypoxic fraction measured using the comet assay and hypoxic fraction measured in the same tumour cells using the paired survival curve assay (slope = 1.20 with 95% confidence limits of 1.03-1.37). These results confirm the ability of the comet assay to provide an accurate estimate of radiobiological hypoxic fraction over a wide range of tumour oxygenations and between two tumour types.


British Journal of Cancer (1997) 76(6), 694–699
© 1997 Cancer Research Campaign

### Detection of hypoxic cells in a C3H mouse mammary carcinoma using the comet assay

**PL Olive[1], MR Horsman[2], C Grau[2] and J Overgaard[2]**

[1]British Columbia Cancer Research Centre, 601 West 10th Avenue, Vancouver, British Columbia, V5Z 1L3 Canada; [2]Department of Experimental Clinical Oncology, Danish Cancer Society, Norrebrogade 44 Aarhus, DK 8000, Denmark

**Summary** The comet assay was used to estimate radiobiological hypoxic fraction across a full range of tumour oxygenations in C3H mammary tumours implanted into the feet of female CDF1 mice. Tumours were either clamped before irradiation or mice were allowed to breath air, 100% oxygen, carbogen or carbon monoxide for 5–35 min before and during exposure to 15 Gy. For the alkaline comet assay, tumours were excised after irradiation and individual tumour cells were analysed for DNA single-strand breaks. Hypoxic cells were defined as those cells with approximately three times fewer single-strand breaks than aerobic cells. Radiobiological hypoxic fraction was calculated by fitting DNA damage histograms to two normal distributions, representing the response of the aerobic and hypoxic populations. The percentage of hypoxic cells estimated using the comet assay was then compared with hypoxic fraction measured using a clamped tumour control assay. Carbogen and oxygen breathing reduced the normal hypoxic fraction from 14% to 2–3% in this tumour, whereas 75–660 p.p.m. carbon monoxide progressively increased the hypoxic fraction from 18% to 82%. The slope of the line comparing the two methods was 1.23 with 95% confidence limits of 1.12–1.33 ($r^2 = 0.994$). In the SCCVII squamous cell carcinoma growing subcutaneously in C3H mice, a similar correlation was observed between hypoxic fraction measured using the comet assay and hypoxic fraction measured in the same tumour cells using the paired survival curve assay (slope = 1.20 with 95% confidence limits of 1.03–1.37). These results confirm the ability of the comet assay to provide an accurate estimate of radiobiological hypoxic fraction over a wide range of tumour oxygenations and between two tumour types.

**Keywords:** tumour hypoxia; DNA strand break; radiosensitivity; tumour control

For over 50 years, hypoxia in solid tumours has been considered to be an important factor capable of limiting the success of conventional radiotherapy (Gray et al, 1953; Bush et al, 1978). As only a proportion of tumours of any one type is now believed likely to contain hypoxic cells at the start of treatment, it is not surprising that clinical trials of hypoxic-cell sensitizers and blood flow modulators have generally shown little benefit when patient numbers entered into trials have been relatively small (Gonzalez, 1991; Dorie and Brown, 1995). When 50 small trials (average patient number = 97) were combined for meta-analysis, however, hypoxic-cell radiosensitizers have demonstrated a benefit in terms of local tumour control (Overgaard, 1994). Ideally, tumours containing hypoxic cells should be identified before treatment in order to direct the appropriate treatments just to those patients who are likely to benefit. To approach this question, a variety of innovative methods have been developed in an effort to measure tumour oxygenation or the presence of hypoxic cells in solid tumours (Stone et al, 1993). Oxygen polarography has been used to measure tumour oxygenation since the late 50s (Cater et al, 1959; Kolstad, 1968; Gatenby et al, 1988), and advances in this technology have made clinical application of this method more reliable and convenient (Vaupel et al, 1991; Nordsmark et al, 1994). Often considered to be the 'gold standard' for measurement of human tumour oxygenation, this technique has undergone the most extensive clinical testing, resulting in demonstration of important correlations between tumour oxygenation and outcome following treatment (Hockel et al, 1993; Nordsmark et al, 1996). Within a single murine tumour type, oxygen electrode measurements correlate with hypoxic fraction measured using conventional assays (Horsman et al, 1993; Nordsmark et al, 1995). However, a limitation identified by several groups is an inability to consistently correlate hypoxic fraction with a specific tumour oxygenation parameter between different tumour types (e.g. median oxygen tension, per cent of readings less than 5 mmHg) (Horsman et al, 1994; Kavanagh et al, 1996). Apparently, the oxygen tension that correlates with radiobiological hypoxia in one type of tumour may differ from the oxygen tension that correlates with hypoxia in another tumour type. This complicates interpretation of oxygen tension measurements made in human tumours, although it should not impact on the ranking of tumours for results obtained by an individual laboratory for a defined histological type.

The comet assay was recently developed as a method for estimating the radiobiological hypoxic fraction (Olive and Durand, 1992; Olive et al, 1993). Radiobiologically hypoxic cells sustain about three times less DNA single-strand breaks than well-oxygenated cells (Chapman et al, 1974; Zhang et al, 1995), forming the basis for detection of individual hypoxic cells from solid tumours. Previous results, using the comet assay in SCCVII murine tumours, indicated good agreement between the hypoxic fraction measured using the comet assay and hypoxic fraction measured using the conventional paired survival curve assay (Olive and Durand, 1992; Olive, 1994).

*Received 2 December 1997*
*Revised 20 February 1997*
*Accepted 27 February 1997*

*Correspondence to:* PL Olive, Medical Biophysics Department, British Columbia Cancer Research Centre, 601 W. 10th Ave., Vancouver, BC, V5Z 1L3, Canada

To examine the ability of the comet assay to measure the hypoxic fraction over a wider range of tumour oxygenations, tumour oxygen tension was increased by allowing mice to breathe oxygen or carbogen, or decreased by breathing air containing various amounts of carbon monoxide (Grau et al, 1994). Using this approach, Horsman et al (1995) showed an excellent relationship between tumour $pO_2$ (both median $pO_2$ and per cent of readings ≤ 5 mmHg) and hypoxic fraction measured using a clamped tumour control end point in a C3H mammary tumour model. All experiments were performed in Aarhus, to allow comparison of results obtained using the comet assay with clamped tumour control data obtained using the C3H mammary tumour in Aarhus.

### METHODS

### Tumour models and irradiations

The majority of experiments were performed on 10 to 14-week-old male CDF1 mice bearing a C3H mouse mammary carcinoma whose derivation and maintenance have been described previously (Overgaard, 1980). Tumours were implanted in the dorsum of the right rear foot and treated when they had reached about 200 $mm^3$ in size. The SCCVII squamous cell carcinoma was grown in the dorsum of the foot (Aarhus) or implanted subcutaneously in the back (Vancouver). SCCVII tumours implanted in the back were used for experiments when they reached a size of 350–500 mg (Olive, 1994). Mice were restrained in lucite jigs and allowed to breathe 100% oxygen or carbogen (95% oxygen, 5% carbon dioxide) for 5 min before and during irradiation, or various percentages of carbon monoxide in air for about 35 min before and during irradiation. The gas flow rate was 2.5 l $min^{-1}$. Foot tumours were made totally hypoxic by clamping the tumour-bearing leg with a rubber band 5 min before and during the irradiation, whereas for back tumours, total hypoxia was achieved using a D-shaped clamp applied for 5 min before and during irradiation. All mice were unanaesthetized and those with foot tumours were exposed to 15 Gy 250 kV X-rays at a dose rate of 2.3 Gy $min^{-1}$, or with back tumours at a dose rate of 3.3 Gy $min^{-1}$.

### Measurement of radiobiological hypoxic fraction

To determine hypoxic fraction, C3H mammary tumours were observed at weekly intervals after treatment and the percentage of animals at each radiation dose showing local tumour control 90 days after irradiation was recorded. Hypoxic fractions were determined from direct analysis of the radiation dose–response data, obtained from clamped or unclamped tumours as described previously (Bentzen and Grau, 1991). Clamped tumours for both tumour control and paired survival curve assays were assumed to contain 100% hypoxic cells. The average number of male mice used to determine each dose–response curve was 235.

For the SCCVII tumour grown in C3H mice, a paired survival curve method was used. Mice were exposed to 12 or 15 Gy while breathing air, carbogen or following asphyxiation. Tumours were immediately excised and divided into two parts. One part was dissociated into single cells using an enzyme cocktail and plated for colony formation (Olive and Durand, 1992). Colonies formed from the surviving cells were counted 12 days later. To determine the hypoxic fraction based on a paired surviving fraction, the clonogenic fraction of cells from the air or carbogen-breathing mouse was divided by the clonogenic fraction of cells from tumours clamped during irradiation. The remaining part of the tumour was used to measure hypoxic fraction using the comet assay as described below.

### Measurement of hypoxic fraction using the comet assay

Six or more tumours were analysed for each gassing condition. Tumours were excised within 30 s of the end of radiation exposure, and were rapidly cooled by submersing in ice-cold phosphate-buffered saline (PBS). They were then chopped with crossed scalpels in ice-cold PBS and filtered through 30-μm nylon mesh. Cells were centrifuged and pellets were resuspended in PBS for dilution to $2 \times 10^4$ cells $ml^{-1}$. More 'background' DNA damage was routinely observed in untreated tumour cells prepared by this method compared with samples prepared using a conventional

**Figure 1** Radiation-induced DNA strand breaks in C3H tumours measured using the comet assay. **A–G** are representative histograms showing distributions of tail moment from individual C3H mammary tumours exposed to 15 Gy while mice breathed different gas mixtures. The percentage of hypoxic cells shown in each is determined using an iterative curve fitting program, assuming two normal distributions for the aerobic and hypoxic cells. **H** shows a typical response of an unirradiated tumour

*© Cancer Research Campaign 1997*

**Figure 2** Comparison between hypoxic fraction measured using the comet assay and hypoxic fraction measured using a clamped tumour control end point for C3H mammary tumours. Results for the comet assay are the means and standard deviations for six to eight tumours. The hypoxic fraction (± 95% confidence intervals) for the clamped tumour control data was determined from full radiation dose–response curves. The average number of male mice used to determine each dose response curve was 235. Lines are the linear best fit and 95% confidence limits for all of the data

**Figure 3** Comparison between mean hypoxic fraction measured using a tumour control assay and two comet descriptors: (**A**) tail moment and (**B**) per cent DNA in comet tail. Means and standard deviations for six to eight tumours, with 400 comets analysed for each tumour are shown. Lines are the linear best-fit through the data and 95% confidence limits enzyme disaggregation procedure in which the tail moment was closer to 2.0. However, this mechanical disaggregation procedure was essential to avoid DNA repair that would occur during enzyme disaggregation at 37°C.

Cell suspensions (0.5 ml) were placed in 5-ml disposable tubes and 1.5 ml of 1% low gelling temperature agarose (Owl Scientific low-gelling agarose prepared in distilled water and held at 40°C) was added to the tube. Then, 1.5 ml was quickly pipetted onto a half-frosted microscope slide and allowed to gel for about 1 min on a cool surface. Slides were carefully submersed in an alkaline lysis solution containing 1.2 M sodium chloride, 0.03 M sodium hydroxide and 0.1% sarkosyl for 1 h, followed by a 1 h wash in 0.03 M sodium hydroxide, 2 mM EDTA before electrophoresis in a fresh solution of 0.03 M sodium hydroxide, 2 mM EDTA at 0.6 volts $cm^{-1}$ for 25 min. Slides were rinsed and stained for 10 min in 2.5 μg $ml^{-1}$ propidium iodide. After rinsing, slides were dried in a 37°C or 50°C oven, and then placed in a light-tight box for transport to Vancouver.

Before analysis, 1 ml of agarose (1%) was pipetted onto the dry slide to reduce background fluorescence for image analysis. Individual cells or 'comets' were viewed using a Zeiss epifluorescence microscope attached to an intensified solid state charge-coupled device camera and image analysis system (Olive et al, 1990). Under these conditions of electrophoresis, as the number of DNA strand breaks increased, the amount of DNA able to migrate away from the comet head increased proportionally to dose. The 'tail moment', defined as the product of the percentage of DNA in the comet tail multiplied by the distance between the means of the head and tail distributions, and 'DNA content', defined as the total fluorescence associated with an image, were measured. Tail moment histograms were obtained from 400 or more comets from the same treated population. Hypoxic fraction was determined by iterative fitting of histograms of tail moment with two normal distributions, representing the aerobic and hypoxic populations (Olive and Durand, 1992; Olive et al, 1993). Slides were randomly coded in Aarhus before analysis in Vancouver.

### RESULTS

Representative tail moment histograms for tumours exposed to 15 Gy are shown in Figure 1A–G. Note the presence of two populations of cells, the less damaged population representing the hypoxic cells of the tumour, and the more damaged population representing the aerobic cells. The curve-fitting program uses a free-fit iterative fitting technique to obtain the best-fit (least sum-of-squares) to the histograms, assuming two normal distributions displaced by a factor varying between 1.9 and 3.0. As expected, the proportion of hypoxic cells decreased as oxygen concentration in the inspired gas increased or as carbon monoxide concentration

© Cancer Research Campaign 1997

**Figure 4** Comparison between hypoxic fraction measured using the comet assay and hypoxic fraction measured using a paired survival curve assay for the SCCVII carcinoma. Means and standard deviations for the SCCVII tumour growing subcutaneously in the back and irradiated in Vancouver (●). Results for the comet assay performed on SCCVII tumours implanted in the foot for air-breathing mice irradiated in Aarhus (▽). Lines are the linear best fit and 95% confidence limits for all of the data decreased. The average displacement between the peaks was 2.1 ± 0.34 (mean ± standard deviation, $n = 40$).

Combined results for six to eight tumours per group are shown in Figure 2 and are compared with the hypoxic fraction measured using a clamped tumour control end point. The slope (± 95% confidence limit) of the line is close to unity (1.2 ± 0.1) and has a correlation coefficient of 0.99.

The correlation between tumour control and either mean tail moment or mean percentage of DNA in the comet tail is also shown (Figure 3). This comparison of average response is not subject to errors in fitting histograms and is similar to tumour hypoxia measurements in which the fraction of hypoxic cells is not specifically detected (i.e. median $pO_2$, average binding of a hypoxia marker).

SCCVII tumours grown subcutaneously in the back of C3H/HeN mice were also examined for hypoxic fraction using the comet assay and compared with results obtained using an in vitro paired survival curve procedure for calculating hypoxic fraction (Figure 4). In addition to experiments performed in Vancouver (closed symbols in Figure 4), one set of six mice with SCCVII tumours growing in the foot was prepared in Aarhus for comet analysis. As expected, hypoxic fraction measured using the comet assay for the smaller tumours grown in the foot in Aarhus was significantly lower ($P$-value < 0.05) than hypoxic fraction measured for the larger subcutaneous SCCVII tumours in Vancouver (0.082 vs 0.18). The value of hypoxic fraction for the foot tumours determined using the comet assay was therefore compared with the previously published value for this tumour growing in the foot (0.002 ± 0.0015) using the paired survival curve method (Grau et al, 1994). The slope of this line was found to be 1.20 with 95% confidence limits of 1.03–1.37.

### DISCUSSION

A good correlation was observed between hypoxic fraction for C3H mammary tumours measured using the comet assay and hypoxic fraction measured using the clamped tumour control end point. As in the oxygen electrode method, the comet assay is able to predict hypoxic fraction in this tumour across the full range of tumour oxygenations. Although the comet assay did underestimate hypoxic fraction, especially in tumours clamped before irradiation, this could be explained by the presence of a small fraction of damaged cells produced during the process of tumour excision and mincing used to prepare a single cell suspension. Experiments with carbon monoxide may be subject to greater interanimal variation, and a smaller variation might have been seen for tumour control experiments performed at the same time as comet experiments. Although it is not possible to perform both comet assay and tumour control experiments on the same mouse, it is possible to perform both comet and in vitro clonogenic assays using cells from the same tumour. This could be an important advantage in the situation in which individual tumour response is variable.

Curve fitting of tail moment histograms for the detection of hypoxic cells becomes less accurate as the proportion of hypoxic or oxic cells is reduced below about 5%. The comparison between tumour control and the raw data obtained using the comet assay (mean tail moment and % DNA in the tail), avoids any bias that might occur in fitting histograms and confirms the ability of the comet assay to detect changes in DNA single-strand breaks that relate to tumour oxygenation (Figure 3). However, in general, raw data are not as useful for measuring tumour hypoxic fraction as results are highly dependent on single-strand break rejoining time during and after irradiation. In contrast, in our experience, hypoxic fraction measured by fitting tail moment histograms (Figure 1) is largely independent of radiation dose over the range of 4–15 Gy, is apparently not affected by repair time (Olive et al, 1994) and is much less dependent on small variations in experimental conditions for the comet assay.

The oxygen electrode method is able to provide an indication of tumour hypoxia across a wide range of tumour oxygenations within a single tumour type. However, there is an apparent inability to correlate $pO_2$ measurements with hypoxic fraction across different tumour types (Horsman et al, 1994; Martin et al, 1994; Kavanagh et al, 1996). Gerwick et al (1995) and Rofstad et al (1988) have also found that tumour adenylate energy charge, NTP/Pi and PCr/Pi ratios do not correlate with radiobiological hypoxia across tumour types, although they are effective indicators within a single tumour type. Nitroimidazole markers, unless analysed at the level of the individual cell, will also produce a signal dependent on nitroreductase activity of the particular tumour, so that the same amount of nitroimidazole binding in different tumour or tissue types cannot be assumed to indicate the same degree of hypoxia (Franko et al, 1987; Cline et al, 1994).

For oxygen electrode measurements, the inability to measure hypoxic fraction reliably across tumour types may result for several reasons. The two most probable explanations are that the fraction of clonogenic hypoxic cells varies for different tumour types (Fenton et al, 1995; Horsman et al, 1995), and that variable degrees of necrosis complicate interpretation of hypoxia (Biade et

© Cancer Research Campaign 1997

al, 1995; Khalil et al, 1995). It is also possible that enzymatic or biochemical factors (unrelated to oxygenation) influence oxygen electrode current readings; recent results indicate that melanin content of cells can influence $pO_2$ measurements (Thomas and Guichard, 1996). Differences in vascular density or sensitivity of blood vessels to damage by electrodes may affect tumour oxygenation unrelated to tumour $pO_2$ (e.g. if bleeding during measurement affects $pO_2$ readings). As the hypoxic fraction of solid tumours depends on the method used to detect hypoxia (Moulder and Rockwell, 1984), the 'true' hypoxic fraction is somewhat illusory. The tumour control assay used for these C3H mammary tumours, perhaps, comes closest to measuring the relevant hypoxic fraction.

For several reasons, intertumour differences are less likely to influence detection of hypoxic cells using the alkaline comet assay:

1. The comet assay measures the response of individual cells, so that necrotic material or heavily damaged cells do not influence the estimation of hypoxic fraction.
2. Cells defined to be hypoxic by this method are radiobiologically hypoxic as the relation between oxygen concentration and DNA damage is the same as the relation between oxygen concentration and cell killing (Chapman et al, 1974; Zhang et al, 1995).
3. The comet assay measures the fraction of radiobiologically hypoxic cells present in tumours at the time of irradiation so that the influence of extraneous conditions should affect the comet assay to the same extent as the tumour control end point.
4. Biochemical factors that might influence estimation of hypoxic fraction using electrodes or hypoxia markers, do not appear to influence the measurement of hypoxic fraction using the comet assay (Zhang and Wheeler, 1994).
5. Whereas the comet assay is invasive, biopsy occurs after the signal (strand breakage) is produced.

Hu et al (1995) have compared the comet assay with Eppendorf oxygen electrode measurements, [$^3$H]misonidazole binding and hypoxic fraction determined using paired survival curve analysis. Using four murine tumours, these authors also concluded that the comet method could detect intertumour differences in hypoxic fraction, although values of hypoxic fraction were two to four times lower than those obtained using the paired survival curve method. Although agreement with [$^3$H]misonidazole binding was reasonable, correlation with oxygen electrode measurements was poor. Unfortunately, the range of tumour oxygenations in experiments by these authors was distributed rather narrowly, making correlations between these methods more difficult. However, our results with two tumour types over a wide range of tumour oxygenation lend further support to the conclusion that intertumour differences can be reliably detected using the comet assay. The ability to obtain information from a single fine-needle aspirate is an important practical advantage, although there is always the concern that the sample may not be representative of the tumour as a whole. Recent analysis of three separate fine-needle aspirates from ten human tumours provides some reassurance on this point (Olive et al, 1996). The comet assay, as in other methods, cannot differentiate between clonogenic and non-clonogenic hypoxic cells. Some differences in hypoxic fraction measured using the comet assay and hypoxic fraction measured using a clonogenic end point would therefore seem inevitable. However, the excellent agreement between the results using the SCCVII tumour and the C3H mammary tumour is encouraging. More serious practical concerns are the requirement of 3.5–4 Gy to be given immediately before fine-needle aspiration biopsy, and the potential influence of circulating white blood cells in the fine-needle aspirate. It is hoped that these are not insurmountable limitations for the routine application of this method in the clinic. Considering the different advantages and disadvantages of the various methods, the comet assay should complement other techniques currently used, or being considered for use, to estimate hypoxic fraction in human solid tumours.

### ACKNOWLEDGEMENTS

The authors gratefully acknowledge the expert technical assistance of IM Thuesen, K Hillebrandt, IM Johansen and C Vikse. This work was supported by NIH grant CA-37879 and by the Danish Cancer Society.

### REFERENCES

Bentzen SM and Grau C (1991) Direct estimation of the fraction of hypoxic cells from tumour-control data obtained under aerobic and clamped conditions. *Inter J Radiat Biol* **59**: 1435–1440

Biade S, Yeh KA, Milito SJ, Brown DQ, Lanciano RM and Chapman JD (1995) Electrode measurements of oxygen tensions in rat prostate carcinomas and comparison with other assays. In *Tumour Oxygenation*, Vaupel P, Kelleher DK and Gunderoth M (eds), pp. 83–94. Gustav Fischer: Stuttgart

Bush RS, Jenkin RD, Allt WE, Beale A, Bean H, Demko AJ and Pringle JF (1978) Definitive evidence for hypoxic cells influencing cure in cancer therapy. *Br J Cancer* **37** (Suppl): 302–306

Cater DB, Silver EA and Wilson BM (1959) Apparatus and technique for the quantitative measurement of oxygen tension in living tissues. *Proc Royal Soc B* **151**: 256–276

Chapman JD, Dugle DL, Reuvers AP, Meeker BE, and Borsa J (1974) Studies on the radiosensitizing effect of oxygen in Chinese hamster cells. *Inter J Radiat Biol* **26**: 383–389

Cline JM, Thrall DE, Rosner GL, and Raleigh JA (1994) Distribution of the hypoxia marker CCI-103F in canine tumours. *Inter J Radiat Oncol Biol Phys* **28**: 921–933

Dorie MJ and Brown JM (1995) Potentiation of the anticancer effect of cisplatin by the hypoxic cytotoxin tirapazamine. In *Tumour Oxygenation*, Vaupel P, Kelleher DK and Gunderoth M, (eds), pp. 125–135. Gustav Fischer: Stuttgart

Fenton BM, Kiani MF and Siemann DW (1995) Should direct measurements of tumour oxygenation relate to the radiobiological hypoxic fraction of a tumour? *Inter J Radiat Oncol Biol Phys* **33**: 365–373

Franko AJ, Koch CJ, Garrecht BM, Sharplin J and Hughes D (1987) Oxygen concentration dependence of binding of misonidazole to rodent and human tumours in vitro. *Cancer Res* **47**: 5367–5376

Gatenby RA, Kessler HB, Rosenblum JS, Coia LR, Moldofsky PJ, Hartz WH and Broder GJ (1988) Oxygen distribution in squamous cell carcinoma metastases and its relationship to outcome of radiation therapy. *Inter J Radiat Oncol Biol Phys* **14**: 831–838.

Gerweck LE, Koutcher J and Zaidi ST (1995) Energy status parameters, hypoxia fraction and radiocurability across tumour types. *Acta Oncol* **34**: 335–338

Gonzalez DG (1991) Hypoxia and local tumour control. Part 1. *Radiother Oncol Suppl* **20**: 5–7

Grau C, Nordsmark M, Khalil AA, Horsman MR and Overgaard J (1994) Effect of carbon monoxide breathing on hypoxia and radiation response in the SCCVII tumor in vivo. *Inter J Radiat Oncol Biol Phys* **29**: 449–454

Gray LH, Conger AD, Ebert M, Hornsey S and Scott OCA (1953) The concentration of oxygen dissolved in tissues at the time of irradiation as a factor in radiotherapy. *Br J Radiol* **26**: 638–648

Höckel M, Knoop C, Schlenger K, Vorndran B, Baubmann E, Mitze M, Knapstein PG and Vaupel P (1993) Intratumoural $pO_2$ predicts survival in advanced cancer of uterine cervix. *Radiother Oncol* **26**: 45–50

Horsman MR, Khalil AA, Nordsmark M, Grau C and Overgaard J (1993) Relationship between radiobiological hypoxia and direct estimates of tumour oxygenation in a mouse tumour model. *Radiother Oncol* **28**: 69–71

© Cancer Research Campaign 1997

Horsman MR, Khalil AA, Siemann DW, Grau C, Hill SA, Lynch EM, Chaplin DJ and Overgaard J (1994) Relationship between radiobiological hypoxia in tumours and electrode measurements of tumour oxygenation. *Inter J Radiat Oncol Biol Phys* **29**: 439–442

Horsman MR, Khalil AA, Nordsmark M, Siemann DW, Hill SA, Lynch M, Chaplin DJ, Stern S, Thomas CD, Guichard M, Grau C and Overgaard J (1995) The use of oxygen electrodes to predict radiobiological hypoxia in tumours. In *Tumour Oxygenation,* Vaupel P, Kelleher DK and Gunderoth M (eds), pp 49–57. Gustav Fischer: Stuttgart

Hu Q, Kavanagh M, Newcombe D and Hill RP (1995) Detection of hypoxic fractions in murine tumours by comet assay: Comparison with other techniques. *Radiat Res* **144**: 266–275

Kavanagh M, Sun A, Hu Q and Hill RP (1996) Comparing techniques of measuring tumour hypoxia in different murine tumours: Eppendorf pO2 histograph, $^{3}$H-misonidazole binding, and paired survival assay. *Radiat Res* **145**: 491–500

Khalil AA, Horsman MR and Overgaard J (1995) The importance of determining necrotic fraction when studying the effect of tumour volume on tissue oxygenation. *Acta Oncologica* **34**: 297–300

Kolstad P (1995) Intercapillary distance, oxygen tension and local recurrence in cervix cancer. *Scand J Clin Lab Invest* **106**: 145–157

Martin LM, Thomas CD and Guichard M (1994) Nicotinamide and carbogen: relationship between pO2 and radiosensitivity in three tumour lines. *Inter J Radiat Biol* **65**: 379–386

Moulder JE and Rockwell S (1984) Hypoxic fractions of solid tumours: experimental techniques, methods of analysis and a survey of existing data. *Inter J Radiat Oncol Biol Phys* **10**: 695–712

Nordsmark M, Bentzen SM and Overgaard J (1994) Measurement of human tumour oxygenation status by a polarographic needle electrode. An analysis of inter- and intratumour heterogeneity. *Acta Oncol* **33**: 383–389

Nordsmark M, Grau C, Horsman MR, Jorgensen HS and Overgaard J (1995) Relationship between tumour oxygenation, bioenergetic status and radiobiological hypoxia in an experimental model. *Acta Oncol* **34**: 329–334

Nordsmark M, Overgaard M and Overgaard J (1996) Pretreatment oxygenation status predicts radiation response in advanced squamous cell carcinoma of the head and neck. *Radiother Oncol* **41**: 31–39

Olive PL (1994) Radiation-induced reoxygenation in the SCCVII murine tumour: evidence for a decrease in oxygen consumption and an increase in tumour perfusion. *Radiother Oncol* **32**: 37–46

Olive PL and Durand RE (1992) Detecting hypoxic cells in a murine tumour using the comet assay. *J Natl Cancer Inst* **85**: 707–711

Olive PL, Banath JP and Durand RE (1990) Heterogeneity in radiation-induced DNA damage and repair in tumour and normal cells measured using the 'comet' assay. *Radiat Res* **122**: 69–72

Olive PL, Durand RE, Le Riche J, Olivotto I and Jackson SM (1993) Gel electrophoresis of individual cells to quantify hypoxic fraction in human breast cancers. *Cancer Res* **53**: 733–736

Olive PL, Vikse, CM and Durand RE (1994) Hypoxic fractions measured in murine tumours and normal tissues using the comet assay. *Inter J Radiat Oncol Biol Phys* **29**: 487–491

Olive PL, Trotter T, Banath JP, Jackson SM and Le Riche J (1996) Heterogeneity in human tumour hypoxic fraction using the comet assay. *Br J Cancer* **74**: S191–S196

Overgaard J (1980) Simultaneous and sequential hyperthermia and radiation treatment of an experimental tumour and its surrounding normal tissue in vivo. *Inter J Radiat Oncol Biol Phys* **6**: 1507–1517

Overgaard J (1994) Clinical evaluation of nitroimidazoles as modifiers of hypoxia in solid tumours. *Oncol Res* **6**: 509–518

Rofstad EK, Demuth P, Fenton BM and Sutherland RM (1988) $^{31}$P-NMR magnetic resonance spectroscopy studies of tumour energy metabolism and its relationship to intracapillary oxyhemoglobin saturation status and tumour hypoxia. *Cancer Res* **48**: 5440–5446

Stone HB, Brown JM, Phillips TM and Sutherland RM (1993) Oxygen in human tumours: correlations between methods of measurement and response to therapy. *Radiat Res* **136**: 422–434

Thomas CD and Guichard M (1996) Influence of melanin on pO2 measurement in vitro and in vivo. *Inter J Radiat Biol* **69**: 205–211

Vaupel P, Schlenger K, Knoop C, and Hockel M (1991) Oxygenation of human tumours: evaluation of tissue oxygen distribution in breast cancers by computerized $O_2$ tension measurements. *Cancer Res* **51**: 3316–3322

Zhang H and Wheeler KT (1994) Radiation-induced DNA damage in tumours and normal tissues. II. Influence of dose, residual DNA damage and physiological factors in oxygenated cells. *Radiat Res* **140**: 321–326

Zhang H, Koch CJ, Wallen CA and Wheeler KT (1995) Radiation-induced DNA damage in tumours and normal tissues. III. Oxygen dependence of the formation of strand breaks and DNA-protein crosslinks. *Radiat Res* **142**: 163–168

© Cancer Research Campaign 1997